# Association of systemic inflammation indices with severe cognitive impairment in Chinese Han patients with Alzheimer’s disease: a cross-sectional study of NLR, PLR, SII, and SIRI

**DOI:** 10.3389/fnagi.2026.1877189

**Published:** 2026-10-27

**Authors:** Yan Wang, Li Lin, Chunxian Xu, Yali Zheng, Quanfeng Zhu

**Affiliations:** 1 Affiliated Xiaoshan Hospital, Hangzhou Normal University, Hangzhou, China; 2 Hangzhou Binjiang District Changhe Subdistrict Community Health Service Center, Hangzhou, China

**Keywords:** Alzheimer’s disease, cognitive impairment, linear relationship, neutrophil-to-lymphocyte ratio, platelet-to-lymphocyte ratio, systemic immune-inflammation index, systemic inflammation indices, systemic inflammation response index

## Abstract

**Objective:**

The relationship between systemic inflammatory markers and cognitive impairment severity in Alzheimer’s disease (AD) remains unclear. This study aimed to investigate the association of the neutrophil-to-lymphocyte ratio (NLR) and additional composite inflammatory indices—platelet-to-lymphocyte ratio (PLR), systemic immune-inflammation index (SII), and systemic inflammation response index (SIRI)—with severe cognitive impairment (SCI) in Chinese Han patients with AD.

**Methods:**

This cross-sectional study included 282 Chinese Han patients with AD. Sociodemographic characteristics and biochemical indicators were collected, and NLR, PLR, SII, and SIRI were calculated. Cognitive function was assessed using the Montreal Cognitive Assessment (MoCA), and SCI was defined as a MoCA score ≤9. Multivariable logistic regression was used to analyze the associations of the four inflammatory indicators with SCI risk. Smooth curve fitting and piecewise linear regression models were applied, with threshold effect analysis performed. Subgroup analyses were conducted according to sex, age, years of education, disease duration, diabetes, hemoglobin, triglycerides, and free triiodothyronine.

**Results:**

After adjustment for covariates, NLR was linearly and positively associated with SCI risk (OR = 1.17, 95% CI: 1.00–1.37, *p* = 0.048). Neither PLR nor SIRI showed a significant association with SCI after multivariable adjustment (*p* > 0.05). In contrast, SII exhibited a non-linear association with SCI risk, with an inflection point at approximately 861.9. Above this threshold, each unit increase in SII was associated with a higher risk of SCI (OR = 1.02, 95% CI: 1.01–1.04, *p* = 0.01), whereas no significant association was observed below the threshold (*p* = 0.06).

**Conclusion:**

In Chinese Han patients with AD, NLR was independently and linearly associated with a higher risk of SCI, while SII showed a threshold effect; PLR and SIRI exhibited no independent associations after multivariable adjustment. Among the four composite inflammatory indices, NLR and SII may hold potential value for assessing cognitive impairment severity in AD, whereas the negative findings for PLR and SIRI suggest that not all composite inflammatory indices are independently associated with SCI.

## Introduction

Alzheimer’s disease (AD) is a neurodegenerative disorder characterized by progressive cognitive decline and behavioral impairments, and represents the most common cause of dementia in the elderly (Zheng and Wang, 2025). With the accelerating aging of the global population, the prevalence of AD continues to rise, posing an increasingly serious public health challenge (Hao and Chen, 2025). AD not only severely compromises patients’ quality of life by progressively depriving them of independent living skills, but also imposes substantial physical and psychological burdens on caregivers, alongside immense socioeconomic and healthcare costs (Chen et al., 2024). Therefore, in-depth investigations into the pathogenesis of AD, along with the identification of effective biomarkers and therapeutic targets, are of critical importance for early detection, disease assessment, and prevention.

In recent years, a growing body of evidence has highlighted the pivotal role of neuroinflammation in the onset and progression of AD (Thakur et al., 2022; Chen and Yu, 2023). Persistent activation of microglia and astrocytes, together with the infiltration of peripheral inflammatory factors, contributes to a complex inflammatory microenvironment within the AD brain. This inflammatory state interacts with hallmark pathologies such as β-amyloid deposition and tau hyperphosphorylation, exacerbating neuronal injury and synaptic dysfunction (Dölling et al., 2022). Both systemic and central nervous system inflammation have been shown to correlate with the rate of cognitive decline in patients with AD (Heneka et al., 2025; Appleton et al., 2025).

Among various inflammatory indicators, composite inflammatory indices derived from routine blood tests have drawn increasing attention owing to their easy accessibility and cost-effectiveness. The neutrophil-to-lymphocyte ratio (NLR), platelet-to-lymphocyte ratio (PLR), systemic immune-inflammation index (SII), and systemic inflammation response index (SIRI) integrate information from distinct immune-inflammatory pathways involving neutrophils, lymphocytes, platelets, and monocytes, and have demonstrated prognostic value in cardiovascular diseases, malignancies, and infectious diseases (Rugambwa et al., 2023; Kawano et al., 2021). Preliminary evidence suggests that elevated peripheral NLR levels may be associated with the risk of AD and global cognitive impairment (Zheng et al., 2025). Whereas studies on PLR, SII, and SIRI in AD remain relatively limited and have yielded inconsistent findings. However, most existing studies have focused on the association between a single inflammatory indicator and the diagnosis of AD or mild cognitive impairment, and studies that systematically compare multiple composite inflammatory indicators in relation to severe cognitive impairment (SCI) in AD are scarce, particularly in the Chinese Han population. Moreover, previous studies have largely assumed a linear relationship between inflammatory indicators and cognitive impairment, with the potential non-linear or threshold effects rarely explored.

Against this background, the present study aimed to evaluate in parallel the associations of NLR, PLR, SII, and SIRI with the severity of cognitive impairment in Chinese Han patients with AD. The novelty of this study lies in simultaneously assessing the associations of four composite inflammatory indicators with SCI in a Chinese Han AD population and characterizing their linear and threshold effects separately, thereby providing more comprehensive evidence for the potential utility of such markers in advanced or rapidly progressing stages of the disease.

## Methods

### Participants

This was a cross-sectional study that enrolled 282 inpatients diagnosed with AD at Affiliated Xiaoshan Hospital, Hangzhou Normal University between January 2020 and December 2025. The inclusion criterion was a diagnosis of AD consistent with the criteria of the National Institute of Neurological and Communicative Disorders and Stroke and the Alzheimer’s disease and Related Disorders Association (NINCDS-ADRDA) (Dubois et al., 2007). The exclusion criteria for the AD group were: (1) cognitive impairment caused by other diseases or conditions, including cognitive impairment due to vascular brain injury; (2) presence of severe concomitant physical illnesses; (3) history of acute infection or use of antibiotics within 1 month prior to enrollment; and (4) autoimmune disorders or use of immunomodulatory drugs within 3 months prior to enrollment. This study was approved by the Ethics Committee of Affiliated Xiaoshan Hospital, Hangzhou Normal University (Approval No. 2024027), and all participants provided written informed consent.

### Collection of demographic, clinical, and laboratory data

Sociodemographic and clinical data collected in this study included sex, age, height, weight, marital status, years of education, smoking status, and alcohol consumption. Additionally, information on each participant’s history of hypertension, history of diabetes, disease duration of AD, use of anti-AD medications, and use of antipsychotic drugs was recorded. Given the potential influence of nonsteroidal anti-inflammatory drugs (NSAIDs) on inflammatory markers, data on NSAID use were also collected (Zádori et al., 2022).

Fasting blood samples were obtained from each participant for laboratory measurements, including high-sensitivity C-reactive protein (hs-CRP); white blood cell (WBC), lymphocyte (LYMPH), neutrophil (NEUT), monocyte (MONO), eosinophil (EO), and basophil (BASO) counts; red blood cell (RBC) count; hemoglobin (HGB); platelet (PLT) count; fasting blood glucose (FBG); triglycerides (TG); total cholesterol (TC); high-density lipoprotein cholesterol (HDL-C); low-density lipoprotein cholesterol (LDL-C); thyroid-stimulating hormone (TSH); free triiodothyronine (FT3); free thyroxine (FT4); total triiodothyronine (T3); and total thyroxine (T4).

The following composite inflammatory indicators were calculated from the above routine blood parameters: NLR = neutrophil count/lymphocyte count; PLR = platelet count/lymphocyte count; SII = platelet count × neutrophil count/lymphocyte count; and SIRI = neutrophil count × monocyte count/lymphocyte count.

Complete blood count parameters were measured using an automated hematology analyzer (model: BC-5180 CRP, Myriad, China). Glucose and lipid profiles were assessed using an automated biochemical analyzer (model: BS-2000, Myriad, China). Thyroid function parameters were measured using an automated chemiluminescence immunoassay analyzer (model: BS-2000, Siemens, Germany). All procedures were performed in accordance with the manufacturers’ instructions.

### Assessment of cognitive function

The Montreal Cognitive Assessment (MoCA) was administered to each participant independently by two trained physicians. The optimized Chinese version of the MoCA has demonstrated sensitivity, specificity, and accuracy exceeding 90% (Jia et al., 2021). The MoCA has a total score of 30 points, with higher scores indicating better cognitive function. The assessments were performed by trained and standardized staff. Based on extensive clinical experience and general consensus in the academic community, a cutoff value most appropriate for the Chinese Han population was adopted in this study, with SCI defined as an MoCA score ≤9.

### Statistical analysis

Missing data were handled using multiple imputation with the following parameters: number of imputations = 20, imputation method = fully conditional specification, maximum iterations = 50, and model type for scale variables = predictive mean matching. Continuous variables with a normal distribution were presented as mean ± standard deviation, while those with a non-normal distribution were presented as median (25th–75th percentile). Categorical variables were described as frequencies and percentages.

After participants were divided into quartiles according to NLR, PLR, SII, and SIRI levels, differences in sociodemographic and clinical parameters among groups were compared using one-way ANOVA, the Kruskal-Wallis *H* test, or the chi-square test, as appropriate. Potential confounders were identified by univariate analysis (*p* < 0.05), and their associations with SCI were assessed. Covariates were selected on the basis of variables with *p* < 0.05 in the between-group comparisons, while variables strongly correlated with one another (e.g., RBC and HGB) were excluded to avoid multicollinearity. Three logistic regression models were constructed separately for each inflammatory indicator: an unadjusted crude model, Model I (adjusted for sex and age), and Model II (adjusted for sex, age, years of education, disease duration, and the metabolic/hematological covariates corresponding to that indicator). Specifically, Model II for NLR was adjusted for sex, age, years of education, disease duration, HGB, TG, and diabetes; Model II for PLR was adjusted for sex, age, years of education, disease duration, body mass index (BMI), FT3, and diabetes; Model II for SII was adjusted for sex, age, years of education, disease duration, HGB, and FT3; and Model II for SIRI was adjusted for sex, age, years of education, disease duration, HGB, TG, PLT, and HDL-C. In each model, the inflammatory indicator was analyzed both as a continuous variable and as a categorical variable (by quartiles). To evaluate model goodness of fit, the Hosmer-Lemeshow test and Nagelkerke *R*^2^ were reported. A Hosmer-Lemeshow test *p*-value > 0.05 indicated good model calibration, and Nagelkerke *R*^2^ was used to assess the ability of the model to explain variation in the outcome.

Smooth curve fitting was performed separately for NLR, PLR, SII, and SIRI to visualize their potential relationships with SCI. Piecewise linear regression models based on generalized estimating equations were used to assess linear or threshold effects; when a non-linear relationship was detected, a recursive algorithm was applied to determine the inflection point. When the log-likelihood ratio test indicated that the non-linear model fit better than the linear model, the inflection point and piecewise ORs were reported. In the threshold effect analysis, the 95% confidence interval of the inflection point was calculated using Bootstrap resampling (1,000 resamples) to evaluate the stability of the threshold estimate. To assess the potential impact of unmeasured confounders on the main results, the *E*-value was calculated. The *E*-value represents the strength of association that an unmeasured confounder would need to have with both the exposure and the outcome to fully explain the observed association to the null (Mathur et al., 2018; VanderWeele and Ding, 2017). A larger *E*-value indicates that the results are more robust to unmeasured confounding.

Subgroup analyses were performed for the indicators significantly associated with SCI in the multivariable models (NLR and SII): NLR was stratified by sex, age, years of education, disease duration, diabetes, HGB, and TG levels; SII was stratified by sex, age, years of education, disease duration, HGB, and FT3 levels. Interaction effects across subgroups were assessed using the log-likelihood ratio test. Subgroup analyses were not performed for PLR or SIRI.

All statistical analyses were performed using R software (http://www.r-project.org, R Foundation) and EmpowerStats (http://www.empowerstats.com, X&Y Solution, Inc., Boston, Massachusetts, United States). A two-tailed *p* < 0.05 was considered statistically significant.

## Results

### Sociodemographic and clinical characteristics of participants

A total of 282 older adult patients were enrolled, ranging in age from 54 to 93 years, including 118 males and 164 females, with a mean age of 75.64 ± 7.65 years. Missing data were distributed as follows: BMI, *n* = 3; FBG, *n* = 2; TG, *n* = 1; TC, *n* = 2; HDL-C, *n* = 1; LDL-C, *n* = 1; TSH, *n* = 3; FT3, *n* = 2; FT4, *n* = 2; T3, *n* = 3; T4, *n* = 2.

The characteristics of the study population stratified by NLR, PLR, SII, and SIRI quartiles are presented in Table 1 and Supplementary Tables S1–S3, respectively. Significant differences were observed among NLR quartiles in WBC, NEUT, LYMPH, MONO, EO, RBC, HGB, TG, sex, and the proportion of patients with diabetes (all *p* < 0.05); among PLR quartiles in BMI, NEUT, LYMPH, EO, PLT, FT3, and the proportion of patients with diabetes (all *p* < 0.05); among SII quartiles in WBC, NEUT, LYMPH, MONO, RBC, HGB, PLT, FT3, and T4 (all *p* < 0.05); and among SIRI quartiles in WBC, NEUT, LYMPH, MONO, RBC, HGB, PLT, CRP, TG, HDL-C, and sex (all *p* < 0.05). The quartile ranges for the four indicators were as follows: NLR Q1 0.56–1.63, Q2 1.64–2.36, Q3 2.36–3.28, and Q4 3.30–16.17; PLR Q1 37.04–103.49, Q2 103.85–140.57, Q3 140.87–186.51, and Q4 190.54–540.00; SII Q1 60.37–288.77, Q2 290.34–451.18, Q3 457.12–668.85, and Q4 676.32–2944.06; and SIRI Q1 0.13–0.50, Q2 0.50–0.76, Q3 0.76–1.19, and Q4 1.21–5.63.

**Table 1:** Baseline characteristics of participants.

Variables	NLR				*p*-value
	Q1 (0.56–1.63)	Q2 (1.64–2.36)	Q3 (2.36–3.28)	Q4 (3.30–16.17)	
N	71	70	70	71	
Age (years old)	75.34 ± 7.41	75.06 ± 7.00	75.29 ± 7.66	76.86 ± 8.46	0.48^a^
Sex (*n*, %)					0.01^b^
Male	26 (36.62)	19 (27.14)	36 (51.43)	37 (52.11)	
Female	45 (63.38)	51 (72.86)	34 (48.57)	34 (47.89)	
Marital status (*n*, %)					0.14^b^
Unmarried	0	2 (2.86)	2 (2.86)	0	
Married	51 (71.83)	42 (60.00)	48 (68.57)	44 (61.97)	
Divorced	0	0	2 (2.86)	0	
Widowed	20 (28.17)	26 (37.14)	18 (25.71)	27 (38.03)	
Hypertension (*n*, %)					0.24^b^
No	25 (35.21)	20 (28.57)	31 (44.29)	29 (40.85)	
Yes	46 (64.79)	50 (71.43)	39 (55.71)	42 (59.15)	
Diabetes (*n*, %)					0.01^b^
No	54 (76.06)	45 (64.29)	55 (78.57)	62 (87.32)	
Yes	17 (23.94)	25 (35.71)	15 (21.43)	9 (12.68)	
NSAIDs (*n*, %)					0.68^b^
No	64 (90.14)	61 (87.14)	60 (85.71)	65 (91.55)	
Yes	7 (9.86)	9 (12.86)	10 (14.29)	6 (8.45)	
Antipsychotic use (*n*, %)					0.43^b^
No	35 (49.30)	38 (54.29)	38 (54.29)	30 (42.25)	
Yes	36 (50.70)	32 (45.71)	32 (45.71)	41 (57.75)	
Anti-AD drugs use (*n*, %)					0.35^b^
No	29 (40.85)	29 (41.43)	38 (54.29)	32 (45.07)	
Yes	42 (59.15)	41 (58.57)	32 (45.71)	39 (54.93)	
Smoking (*n*, %)					0.25^b^
No	60 (84.51)	60 (85.71)	52 (74.29)	55 (77.46)	
Yes	11 (15.49)	10 (14.29)	18 (25.71)	16 (22.54)	
Alcohol consumption (*n*, %)					0.77^b^
No	55 (77.46)	55 (78.57)	50 (71.43)	54 (76.06)	
Yes	16 (22.54)	15 (21.43)	20 (28.57)	17 (23.94)	
SCI (*n*, %)					0.14^b^
No	41 (57.75)	34 (48.57)	40 (57.14)	29 (40.85)	
Yes	30 (42.25)	36 (51.43)	30 (42.86)	42 (59.15)	
BMI (kg/m^2^)	21.88 ± 3.55	22.27 ± 3.39	21.87 ± 3.53	21.17 ± 3.31	0.29^a^
Years of education (years)	0 (0–6.00)	0 (0–6.00)	1.00 (0–6.00)	0 (0–6.00)	0.94^c^
Disease duration (years)	3.00 (2.00–5.00)	3.00 (2.00–5.00)	3.50 (2.00–5.00)	3.00 (2.00–5.00)	0.87^c^
MoCA score	11.30 ± 4.98	9.51 ± 4.47	10.67 ± 5.60	9.93 ± 5.53	0.18^a^
WBC (10^^^9/L)	4.67 ± 1.16	5.15 ± 1.35	5.06 ± 1.56	6.39 ± 1.73	<0.001^a^
NEUT (10^^^9/L)	2.36 ± 0.68	3.13 ± 0.87	3.39 ± 1.03	4.89 ± 1.55	<0.001^a^
LYMPH (10^^^9/L)	1.85 ± 0.53	1.53 ± 0.40	1.24 ± 0.37	1.00 ± 0.27	<0.001^a^
MONO (10^^^9/L)	0.31 ± 0.08	0.35 ± 0.12	0.34 ± 0.12	0.40 ± 0.15	<0.001^a^
EO (10^^^9/L)	0.13 (0.08–0.17)	0.10 (0.07–0.17)	0.08 (0.06–0.14)	0.07 (0.03–0.14)	<0.001^c^
BASO (10^^^9/L)	0.01 (0.01–0.02)	0.01 (0.01–0.02)	0.01 (0.01–0.02)	0.01 (0.01–0.02)	0.12^c^
RBC (10^^^12/L)	3.92 ± 0.54	3.95 ± 0.50	4.02 ± 0.44	4.14 ± 0.46	0.03^a^
HGB (g/L)	118.75 ± 19.18	121.71 ± 13.28	124.21 ± 13.77	126.87 ± 14.10	0.01^a^
PLT (10^^^9/L)	183.99 ± 59.96	195.14 ± 59.05	206.23 ± 83.74	202.70 ± 50.63	0.18^a^
hs-CRP (mg/L)	1.34 (0.80–2.49)	1.75 (0.76–3.80)	1.94 (1.11–3.73)	1.91 (0.86–3.73)	0.10^c^
FBG (mmol/L)	5.27 ± 1.99	5.42 ± 1.51	5.29 ± 1.64	5.56 ± 1.36	0.70^a^
TG (mmol/L)	1.32 ± 0.79	1.31 ± 0.76	1.16 ± 0.54	1.01 ± 0.53	0.02^a^
TC (mmol/L)	4.20 ± 0.86	4.20 ± 1.07	4.02 ± 0.85	4.31 ± 0.93	0.32^a^
HDL-C (mmol/L)	1.21 ± 0.31	1.28 ± 0.74	1.18 ± 0.29	1.35 ± 0.41	0.17^a^
LDL-C (mmol/L)	2.68 ± 0.77	2.71 ± 0.88	2.56 ± 0.72	2.67 ± 0.73	0.71^a^
TSH (uIU/mL)	1.93 (1.40–2.96)	1.71 (1.14–2.57)	1.94 (1.18–2.83)	1.97 (1.24–2.94)	0.71^c^
FT3 (pmol/L)	3.10 ± 0.41	3.03 ± 0.57	3.06 ± 0.47	2.98 ± 0.49	0.54^a^
FT4 (pmol/L)	1.15 ± 0.19	1.17 ± 0.25	1.20 ± 0.23	1.22 ± 0.24	0.33^a^
T3 (nmol/L)	1.01 ± 0.23	1.03 ± 0.20	1.07 ± 0.22	1.11 ± 0.42	0.16^a^
T4 (nmol/L)	7.63 ± 2.11	8.26 ± 2.39	8.13 ± 1.94	8.50 ± 2.18	0.11^a^

The variables are presented as *n* (%) or the mean ± SD or median (quartile 1-quartile 3).

NLR, neutrophil/lymphocyte ratio; NSAIDs, nonsteroidal antiinflammatory drugs; AD, Alzheimer’s disease; SCI, severe cognitive impairment; BMI, body mass index; MoCA, Montreal Cognitive Assessment; WBC, white blood cell; NEUT, neutrophil; LYMPH, lymphocyte; MONO, monocytes; EO, eosinophil; BASO, basophil; RBC, red blood cell; HGB, hemoglobin; PLT, platelet; hs-CRP, hypersensitive C-reactive protein; FBG, fasting blood glucose; TG, triglycerides; TC, total cholesterol; HDL-C, high-density lipoprotein cholesterol; LDL-C, low-density lipoprotein cholesterol; TSH, thyroid stimulating hormone; FT3, free triiodothyronine; FT4, free thyroxine; T3, triiodothyronine; T4, thyroxine.

^a^one-way ANOVA test, ^b^*χ*2 test, and ^c^Kruskal–Wallis *H* test.

### Association between systemic inflammation indices and SCI

As shown in Table 2, the univariate analysis revealed that NLR (OR = 1.20, 95% CI: 1.04–1.39, *p* = 0.01), SII (OR = 1.00, 95% CI: 1.00–1.00, *p* = 0.03), and SIRI (OR = 1.36, 95% CI: 1.03–1.80, *p* = 0.03) were significantly associated with SCI, whereas the association between PLR and SCI did not reach statistical significance (OR = 1.00, 95% CI: 1.00–1.01, *p* = 0.11).

**Table 2:** The result of univariate analysis for severe cognitive impairment.

Covariate	Statistics	OR (95%CI)	*p*-value
NLR	2.82 ± 1.87	1.20 (1.04, 1.39)	0.01
PLR	156.30 ± 72.62	1.00 (1.00, 1.01)	0.11
SII	565.44 ± 431.14	1.00 (1.00, 1.00)	0.03
SIRI	1.04 ± 0.89	1.36 (1.03, 1.80)	0.03

OR, odds ratio; CI, confidence interval; NLR, neutrophil-to-lymphocyte ratio; PLR, platelet-to-lymphocyte ratio; SII, systemic immune-inflammation index; SIRI, systemic inflammation response index.

Table 3 presents the associations between the four inflammatory indicators and SCI after adjustment for confounders, as detailed below:

**Table 3:** Relationship between systemic inflammation indices (NLR, PLR, SII, SIRI) and severe cognitive impairment in different models.

Variable	Unadjusted Model		Model I		Model II	
	OR (95% CI)	*p*-value	OR (95% CI)	*p*-value	OR (95% CI)	*p*-value
NLR	1.20 (1.04, 1.39)	0.01	1.19 (1.03, 1.38)	0.02	1.17 (1.00, 1.37)	0.048
NLR (quartile)						
Q1	1.00 (Ref)		1.00 (Ref)		1.00 (Ref)	
Q2	1.45 (0.74, 2.81)	0.28	1.45 (0.74, 2.83)	0.28	1.54 (0.76, 3.10)	0.23
Q3	1.03 (0.53, 2.00)	0.94	1.04 (0.53, 2.05)	0.90	0.96 (0.47, 1.93)	0.90
Q4	1.98 (1.02, 3.86)	0.045	1.95 (0.99, 3.83)	0.054	1.74 (0.85, 3.55)	0.13
*p* for trend		0.11		0.12		0.30
PLR	1.00 (1.00, 1.01)	0.11	1.00 (1.00, 1.01)	0.12	1.00 (1.00, 1.01)	0.21
PLR (quartile)						
Q1	1.00 (Ref)		1.00 (Ref)		1.00 (Ref)	
Q2	0.92 (0.47, 1.78)	0.80	0.95 (0.49, 1.86)	0.89	0.93 (0.47, 1.87)	0.85
Q3	0.82 (0.42, 1.59)	0.55	0.81 (0.41, 1.58)	0.53	0.92 (0.46, 1.84)	0.82
Q4	1.58 (0.81, 3.06)	0.18	1.60 (0.82, 3.13)	0.17	1.49 (0.73, 3.01)	0.27
*p* for trend		0.24		0.25		0.31
SII	1.00 (1.00, 1.00)	0.03	1.00 (1.00, 1.00)	0.03	1.00 (1.00, 1.00)	0.03
SII (quartile)						
Q1	1.00 (Ref)		1.00 (Ref)		1.00 (Ref)	
Q2	0.87 (0.45, 1.68)	0.67	0.88 (0.45, 1.71)	0.70	0.89 (0.45, 1.76)	0.75
Q3	0.69 (0.35, 1.34)	0.27	0.71 (0.36, 1.40)	0.33	0.71 (0.35, 1.41)	0.33
Q4	1.58 (0.81, 3.07)	0.18	1.63 (0.83, 3.19)	0.15	1.66 (0.83, 3.32)	0.15
*p* for trend		0.29		0.25		0.26
SIRI	1.36 (1.03, 1.80)	0.03	1.35 (1.01, 1.80)	0.04	1.32 (0.97, 1.80)	0.08
SIRI (quartile)						
Q1	1.00 (Ref)		1.00 (Ref)		1.00 (Ref)	
Q2	1.45 (0.75, 2.81)	0.27	1.52 (0.78, 2.96)	0.22	1.40 (0.69, 2.83)	0.35
Q3	0.86 (0.44, 1.68)	0.66	0.85 (0.43, 1.68)	0.65	0.87 (0.42, 1.82)	0.72
Q4	1.49 (0.77, 2.88)	0.24	1.49 (0.76, 2.92)	0.25	1.27 (0.61, 2.65)	0.52
*p* for trend		0.52		0.54		0.82

Unadjusted model: no covariates adjusted; Model I adjusted for age and sex. Model II for NLR adjusted for age, sex, years of education, disease duration, HGB, TG, and diabetes; Model II for PLR adjusted for age, sex, years of education, disease duration, BMI, FT3, and diabetes; Model II for SII adjusted for age, sex, years of education, disease duration, HGB, and FT3; Model II for SIRI adjusted for age, sex, years of education, disease duration, HGB, TG, PLT, and HDL-C. OR, odds ratio; CI, confidence interval; NLR, neutrophil-to-lymphocyte ratio; PLR, platelet-to-lymphocyte ratio; SII, systemic immune-inflammation index; SIRI, systemic inflammation response index.

NLR: In Model I (adjusted for sex and age), NLR was significantly and positively associated with SCI (OR = 1.19, 95% CI: 1.03–1.38, *p* = 0.02); this association persisted in Model II (adjusted for sex, age, years of education, disease duration, HGB, TG, and diabetes) (OR = 1.17, 95% CI: 1.00–1.37, *p* = 0.048). When analyzed as a categorical variable, only the unadjusted model showed a significantly higher probability of SCI in Q4 than in Q1 (*p* < 0.05); no significant differences across quartiles were observed in the adjusted models (all *p* > 0.05).

PLR: Neither as a continuous variable nor as a quartile-based categorical variable was PLR significantly associated with SCI in the unadjusted model, Model I, or Model II (Model II: OR = 1.00, 95% CI: 1.00–1.01, *p* = 0.21; Q4 vs. Q1: OR = 1.49, 95% CI: 0.73–3.01, *p* = 0.27).

SII: As a continuous variable, SII was significantly associated with SCI in the unadjusted model (OR = 1.00, 95% CI: 1.00–1.00, *p* = 0.03), Model I (*p* = 0.03), and Model II (*p* = 0.03); however, when analyzed as a quartile-based categorical variable, no significant differences were found across quartiles (all *p* > 0.05).

SIRI: SIRI was significantly associated with SCI in the unadjusted model (OR = 1.36, 95% CI: 1.03–1.80, *p* = 0.03) and Model I (OR = 1.35, 95% CI: 1.01–1.80, *p* = 0.04); however, after adjustment for sex, age, years of education, disease duration, HGB, TG, PLT, and HDL-C in Model II, the association was no longer significant (OR = 1.32, 95% CI: 0.97–1.80, *p* = 0.08).

The goodness-of-fit tests for the multivariable logistic regression models showed that the NLR model was significant overall (Omnibus *χ*^2^ = 25.66, df = 8, *p* = 0.001), with a Hosmer-Lemeshow test *p* = 0.663 and Nagelkerke *R*^2^ = 0.116, indicating good calibration and moderate explanatory ability. The SII model was also significant overall (Omnibus *χ*^2^ = 18.64, df = 7, *p* = 0.01), with a Hosmer-Lemeshow test *p* = 0.79 and Nagelkerke *R*^2^ = 0.09, indicating acceptable model fit.

Smooth curve fitting showed a linear relationship between NLR and the predicted probability of SCI (s(NLR) edf = 1.0003, *p* = 0.048) (Figure 1); the smooth terms for PLR and SIRI were not significant (*p* = 0.24 and *p* = 0.14, respectively); and the smooth term for SII approached but did not reach statistical significance (edf = 3.45, *p* = 0.06), suggesting a possible non-linear relationship.

**Figure 1:**
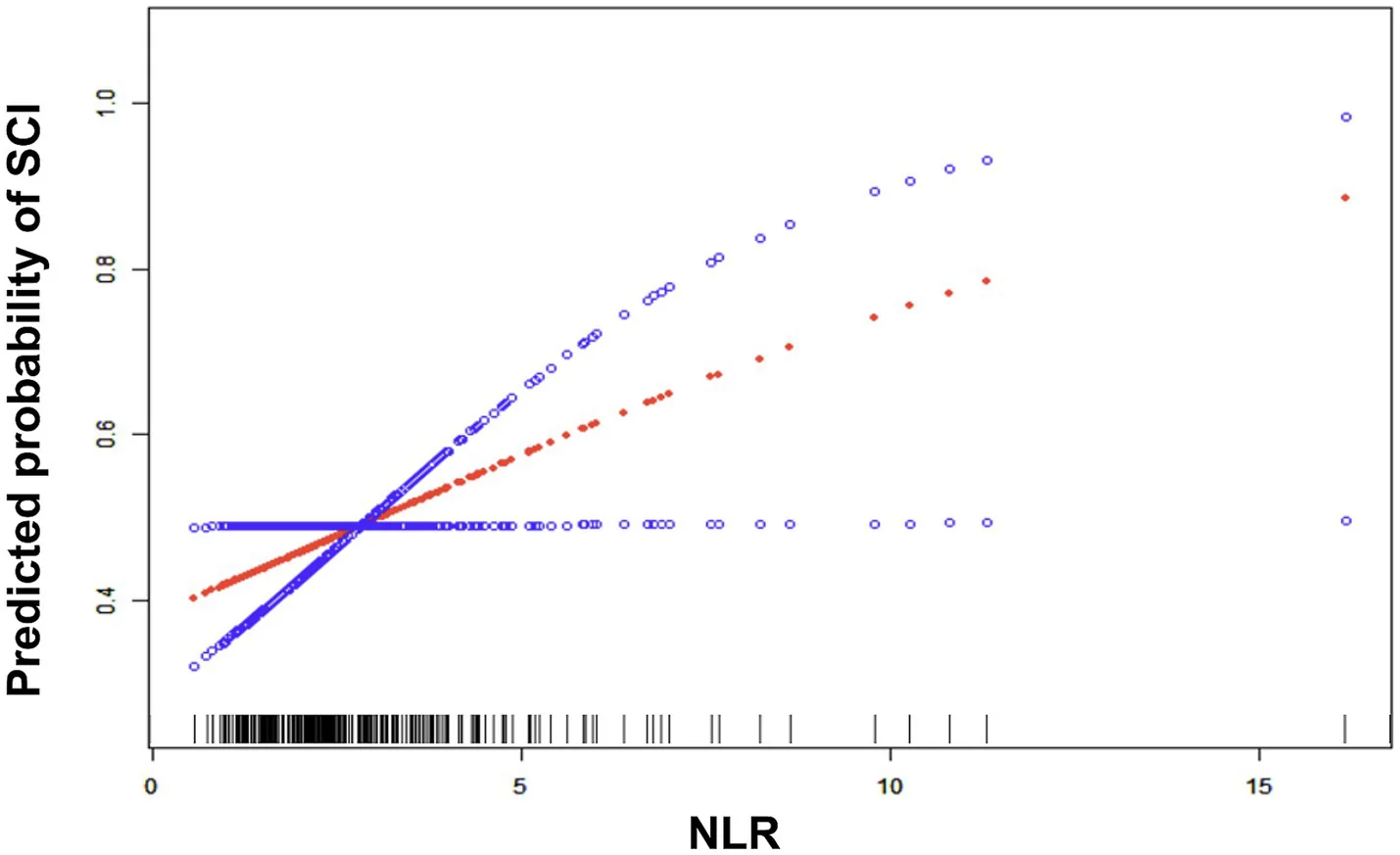
The relationship between NLR and the predicted probability of SCI. The predicted probability of SCI was derived from a multivariable logistic regression model adjusted for age, sex, years of education, disease duration, HGB, TG, and history of diabetes (Model II in Table 3). The red dotted curve represents the non-linear trend fitted by smooth curve fitting, and the upper and lower blue dotted curves represent the 95% confidence intervals. The rug plot along the x-axis shows the distribution of NLR values. The analysis included 282 Chinese Han patients with AD. NLR, neutrophil-to-lymphocyte ratio; SCI, severe cognitive impairment; AD, Alzheimer’s disease; HGB, hemoglobin; TG, triglycerides.

Accordingly, threshold effect analysis was further performed for NLR and SII (Table 4). For NLR, the log-likelihood ratio test of the piecewise model yielded *p* = 0.32, indicating that the linear model was superior, consistent with the smooth curve fitting results. For SII, the non-linear model was superior to the linear model (log-likelihood ratio test *p* = 0.01), with an inflection point at approximately 861.9 (95% CI: 653.01–915.34); below the inflection point, the association between SII and SCI was not significant (*p* = 0.06), whereas above the inflection point, each unit increase in SII was associated with a higher risk of SCI (OR = 1.02, 95% CI: 1.01–1.04, *p* = 0.01), and the difference in effects on either side of the inflection point was statistically significant (*p* = 0.01). For SIRI, the log-likelihood ratio test of the piecewise model yielded *p* = 0.06, which did not reach statistical significance. Because the smooth terms for PLR and SIRI were not significant, no further threshold effect analysis was performed for these two indicators.

**Table 4:** The results of two-piecewise logistic regression model.

Exposure	Inflection point	Effect size (OR)	95% CI	*p*-value
NLR	<3.08	0.97	0.66, 1.44	0.90
	≥3.08	1.28	1.00, 1.65	0.054
	Log-likelihood			0.32
	95% CI for inflection point		2.53, 3.76	
SII	<861.9	1.00	1.00, 1.00	0.06
	≥861.9	1.02	1.01, 1.04	0.01
	Log-likelihood			0.01
	95% CI for inflection point		653.01, 915.34	

Outcome: SCI; Exposure: NLR/SII; Model for NLR adjusted for age, sex, years of education, disease duration, HGB, TG, and diabetes; Model for SII adjusted for age, sex, years of education, disease duration, HGB, and FT3. OR, odds ratio; CI, confidence interval; NLR, neutrophil-to-lymphocyte ratio; SII, systemic immune-inflammation index.

Sensitivity analysis showed that the point-estimate *E*-value for the association between NLR and SCI (Model II: OR = 1.17, 95% CI: 1.00–1.37) was 1.63, and the *E*-value for the lower confidence interval limit was 1.02, suggesting that this association was somewhat robust to unmeasured confounding, although its statistical significance was borderline. The point-estimate *E*-value for SII was 1.01, indicating that its association was relatively sensitive to unmeasured confounding.

### Subgroup analyses

Subgroup analyses (Figures 2, 3) showed that the association between NLR and SCI was consistent across subgroups (all *p*-values for interaction >0.05). In the subgroup analyses of SII, the *p*-values for interaction were all >0.05 (sex, *p* = 0.96; age, *p* = 0.10; disease duration, *p* = 0.53; years of education, *p* = 0.54; HGB, *p* = 0.93; FT3, *p* = 0.97), indicating no significant heterogeneity in the association between SII and SCI across subgroups. Subgroup analyses of PLR and SIRI did not reveal significant subgroup interaction effects.

**Figure 2:**
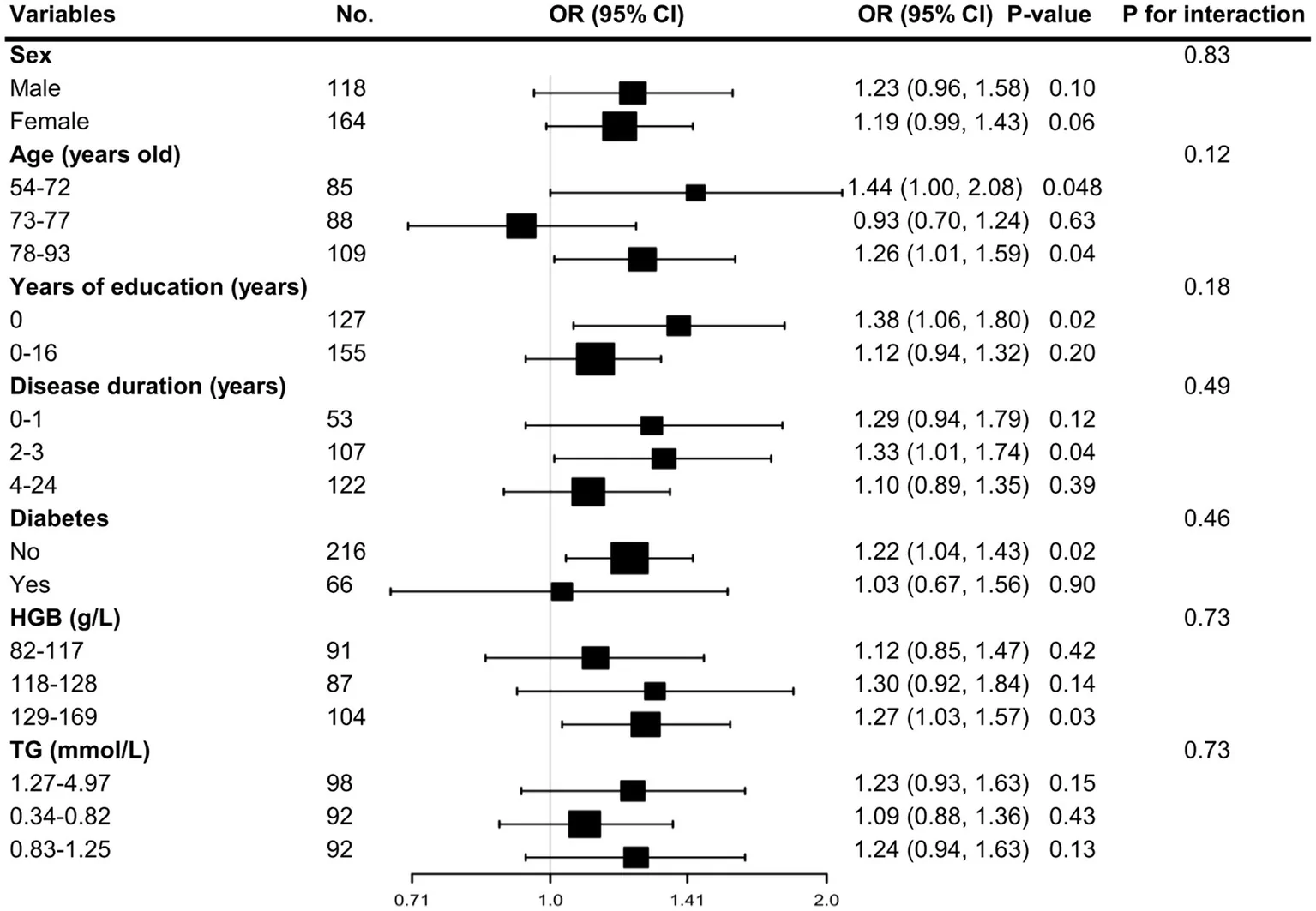
Subgroup analysis of the association between NLR and SCI. The OR (95% CI) for each subgroup was derived from multivariable logistic regression models. The analysis was stratified by sex (male, female), age (54–72, 73–77, 78–93 years), years of education (6–16, 0–1 years), disease duration (0–1, 2–3, 4–24 years), diabetes (no, yes), HGB (82–117, 118–128, 129–169 g/L), and TG levels (0.34–0.82, 0.83–1.25, 1.27–4.97 mmol/L). The model was adjusted for age, sex, years of education, disease duration, HGB, TG, and diabetes, except that the stratification variable itself was not included as a covariate in the corresponding subgroup model. The *p*-value for interaction was calculated using the log-likelihood ratio test. The size of the squares reflects the weight of each subgroup, and the horizontal lines represent the 95% CIs. NLR, neutrophil-to-lymphocyte ratio; SCI, severe cognitive impairment; OR, odds ratio; CI, confidence interval; HGB, hemoglobin; TG, triglycerides.

**Figure 3:**
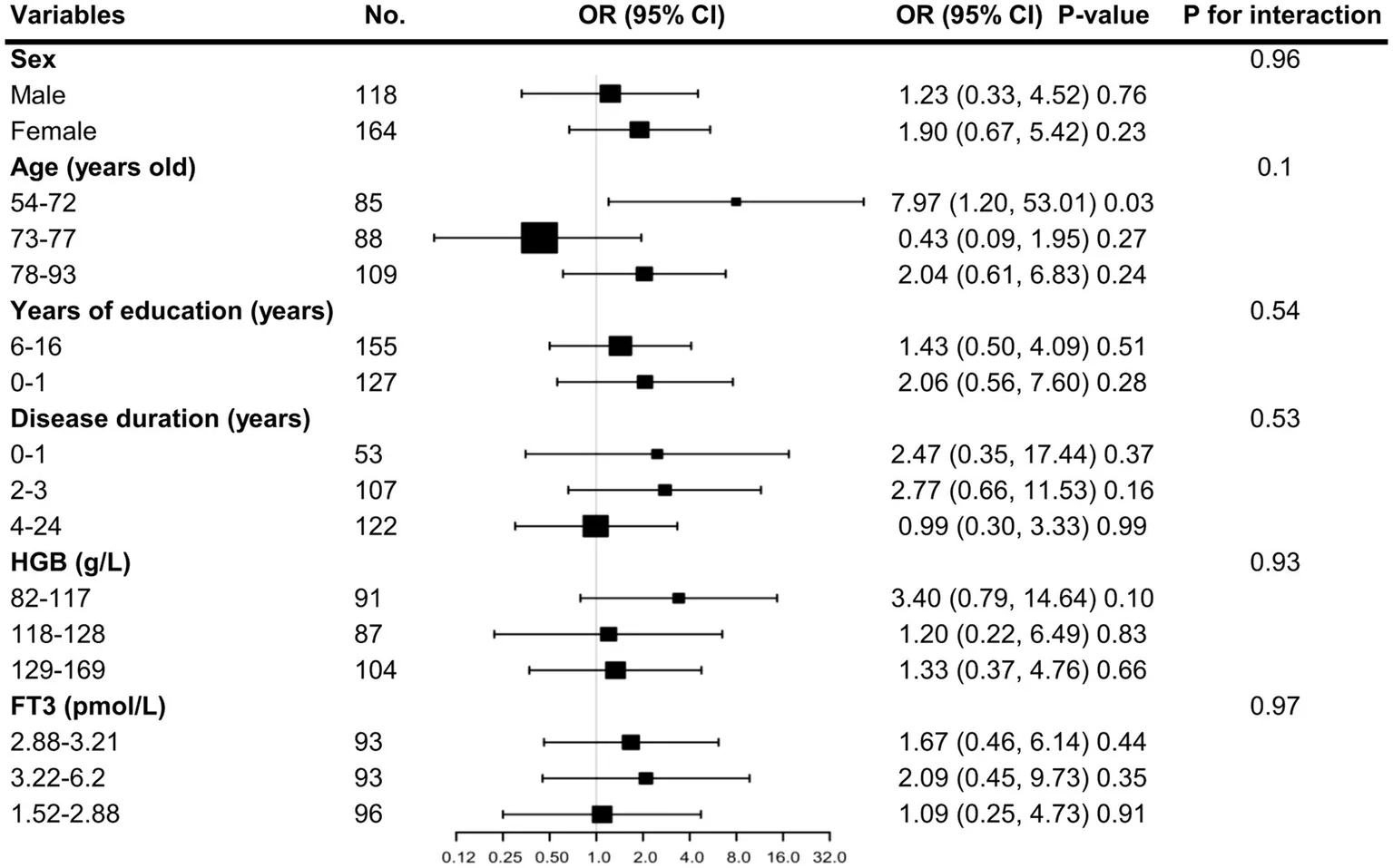
Subgroup analysis of the association between SII and SCI. The OR (95% CI) for each subgroup was derived from multivariable logistic regression models. SII was log10-transformed for analysis, and the ORs are expressed per 1-unit increase in log10(SII). The analysis was stratified by sex (male, female), age (54–72, 73–77, 78–93 years), years of education (6–16, 0–1 years), disease duration (0–1, 2 3, 4–24 years), HGB (82–117, 118–128, 129–169 g/L), and FT3 levels (2.88–3.21, 3.22–6.2, 1.52–2.88 pmol/L). The model was adjusted for age, sex, years of education, disease duration, HGB, and FT3, except that the stratification variable itself was not included as a covariate in the corresponding subgroup model. The *p*-value for interaction was calculated using the log-likelihood ratio test. The size of the squares reflects the weight of each subgroup, and the horizontal lines represent the 95% CIs. SII, systemic immune-inflammation index; SCI, severe cognitive impairment; OR, odds ratio; CI, confidence interval; HGB, hemoglobin; FT3, free triiodothyronine.

## Discussion

In this cross-sectional study of 282 Chinese Han patients with AD, we evaluated in parallel the associations of four composite inflammatory indices—NLR, PLR, SII, and SIRI—with the risk of severe cognitive impairment. The results showed that NLR was independently and linearly associated with a higher risk of SCI; SII exhibited a threshold effect on SCI risk (with an inflection point at approximately 861.9); PLR showed no independent association after multivariable adjustment; and the association for SIRI weakened to borderline non-significance after full adjustment. These findings suggest that not all composite inflammatory indices are independently associated with the severity of cognitive impairment in patients with AD. It should be emphasized that, given the cross-sectional design of this study, no causal direction can be inferred; the interpretations below are therefore hypothetical and require validation through mechanistic studies and prospective cohorts.

A major feature of this study is the parallel comparison of four routine blood-derived composite inflammatory indices. Both NLR and SII incorporate neutrophil and lymphocyte counts, but SII additionally integrates platelet count. In this study, NLR showed a linear positive association, whereas SII exhibited a threshold effect, suggesting that the inclusion of the platelet component may alter the shape of the association between the indicator and SCI. PLR integrates only platelets and lymphocytes, without neutrophils, and showed no independent association in this study, implying that neutrophil-related pathways may be more important than the platelet–lymphocyte balance alone in cognitive impairment at advanced stages of AD. SIRI integrates neutrophils, monocytes, and lymphocytes; it was associated with SCI in the unadjusted model and in the model adjusted only for sex and age, but the association weakened after full adjustment for metabolic and hematological covariates such as HGB, TG, PLT, and HDL-C, suggesting that its effect may be partly mediated by metabolic/hematological factors or limited by sample size. These discrepancies suggest that future studies should select composite inflammatory indices in light of the specific disease stage and pathological mechanisms, rather than simply assuming that these indicators are interchangeable.

The findings of this study are consistent with a growing body of literature on the association between peripheral blood inflammatory markers and cognitive function in AD (Hou et al., 2022; Xia et al., 2025). Multiple studies have demonstrated that NLR levels are significantly higher in AD patients than in healthy controls and are associated with worse cognitive performance (Hou et al., 2022; Mehta et al., 2023; Cervellati et al., 2024; Dong et al., 2019; Mohammadi et al., 2024). However, most existing evidence has focused on comparing NLR differences between AD patients and cognitively normal individuals, or has been conducted in mixed cohorts including various types of cognitive impairment (e.g., mild cognitive impairment, vascular dementia) (Toubasi and Al-Sayegh, 2026). In contrast, the present study focused specifically on patients with confirmed AD and adopted a more stringent outcome—severe cognitive impairment (MoCA score ≤9)—thereby further clarifying that, among patients with confirmed AD, an independent positive association exists between NLR levels and the severity of cognitive impairment (OR = 1.17, 95% CI: 1.00–1.37, *p* = 0.048 after adjustment for sex, age, years of education, disease duration, HGB, TG, and diabetes). Compared with NLR, PLR, SII, and SIRI have been less studied in AD. In previous mixed cognitive impairment cohorts or cross-sectional studies, SII and SIRI have been reported to be associated with cognitive decline, although the findings have been inconsistent (Guo et al., 2023; Zhang et al., 2026; Tondo et al., 2023). In the present study of Chinese Han patients with AD, no independent association was found for PLR after multivariable adjustment, the association for SIRI also weakened to borderline non-significance, and SII exhibited a threshold effect, which may be attributable to differences across studies in population, disease stage, covariate adjustment, and modeling approach.

In terms of statistical modeling, although the linear model for SII in this study yielded a significant *p*-value, the quartile-based model did not show a significant trend, suggesting that its effect may not follow a simple dose–response relationship but rather a non-linear/threshold pattern. This is consistent with the findings of the threshold effect analysis (inflection point at approximately 861.9; above the inflection point, OR = 1.02, *p* = 0.0107; below the inflection point, *p* = 0.0592). This finding underscores the importance of simultaneously reporting continuous-variable, categorical-variable, and smooth curve/threshold analyses in studies of inflammatory markers: relying on a linear model alone or on a quartile-based model alone may both lead to missing or misinterpreting the true shape of the association.

Furthermore, our results remained robust after adjusting for several metabolic parameters, including TG and history of diabetes, in the multivariate models. Metabolic disturbances, particularly insulin resistance and lipid abnormalities, are closely associated with the onset and progression of AD, and their pathogenic mechanisms also involve activation of inflammatory pathways (Kellar and Craft, 2020; Song et al., 2025; Skawratananond et al., 2025). In this study, after accounting for the influence of significantly different metabolic parameters, NLR remained independently associated with severe cognitive impairment, suggesting that the inflammatory burden reflected by NLR may be a risk factor for cognitive impairment independent of conventional metabolic factors. The negative association between TG levels and SCI observed in the univariate analysis (OR = 0.53, *p* = 0.002) should be interpreted with caution: this analysis did not adjust for covariates, and the cross-sectional design precludes any inference regarding causal direction. This phenomenon may be related to metabolic alterations in advanced AD, such as reduced blood lipid levels resulting from weight loss and deteriorating nutritional status, but prospective studies are needed for validation (Inamdar et al., 2025; Dimache et al., 2021).

The clinical implications of this study should be interpreted in light of the fact that cognitive severity in AD is already well captured by neuropsychological instruments such as the MoCA. The value of NLR and SII therefore does not lie in replacing cognitive assessment, but in providing complementary information on systemic inflammatory burden that is not reflected by cognitive scores alone. NLR remained independently associated with SCI after multivariable adjustment, suggesting that it may help identify, among patients with comparable cognitive scores, those with a higher systemic inflammatory burden who might warrant closer follow-up. The threshold effect of SII indicates that once SII exceeds a certain level, clinicians may consider this a signal of increased inflammatory load; however, whether this translates into prognostic value requires validation in longitudinal cohorts with repeated cognitive assessments and clinical outcomes. The negative findings for PLR and SIRI further suggest that not all composite inflammatory indices are equally informative, and that their clinical interpretation should be tied to the specific index and disease stage. Future studies should focus on whether these indices predict cognitive decline, conversion, or other clinically meaningful outcomes, rather than merely correlating with cross-sectional severity.

This study has several limitations. First, the cross-sectional design allowed us to examine only the associations between inflammatory indicators and the severity of cognitive impairment, precluding any inference of causality. That is, it remains unclear whether a higher inflammatory burden leads to more severe cognitive impairment or whether a severely impaired cognitive state (for example, accompanied by malnutrition and reduced physical activity) leads to elevated inflammatory indicators. Future prospective cohort studies are needed to clarify the temporal sequence. Second, severe cognitive impairment was defined in this study as a MoCA score ≤9, and the choice of this threshold warrants explanation among the limitations. The MoCA was originally designed for mild cognitive impairment screening and is widely used in community and clinical settings, but its applicability for assessing the severity of cognitive impairment in patients with AD remains controversial. The severity of cognitive symptoms in AD is more commonly assessed with dedicated instruments such as the Alzheimer’s disease Assessment Scale–Cognitive Subscale (ADAS-Cog) (Kueper et al., 2018). The use of MoCA ≤9 as the criterion for severe cognitive impairment in this study was based primarily on its widespread use in Chinese populations and its clinical operability; however, this threshold may not be fully equivalent to the severe stage as defined by ADAS-Cog. In addition, the MoCA covers a relatively limited range of cognitive domains and does not assess specific domains such as executive function, language, and visuospatial ability. Future studies employing more comprehensive neuropsychological batteries such as ADAS-Cog or neurophysiological methods are needed to further validate our findings and to explore the relationships between inflammatory indicators and impairment in different cognitive domains. Third, all participants in this study were Han Chinese patients with AD from a single center, and the generalizability of our findings to other ethnic groups, populations, or community-dwelling patients with AD requires further validation. Moreover, NLR, PLR, SII, and SIRI are all composite indicators derived from routine blood tests and are susceptible to factors such as acute infection, medications, and the timing of blood collection; although we excluded individuals with a history of acute infection or antibiotic use within 1 month before enrollment, subclinical infection or short-term medication effects on these indicators could not be fully ruled out. In addition, some collinearity exists among the four indicators (for example, both NLR and SII include neutrophil and lymphocyte counts), and future studies may consider approaches such as joint models or principal component analysis to further explore their independent and combined effects. Fourth, despite adjustment for several important confounders, as an observational study it may still be affected by unmeasured confounders (for example, individual genetic polymorphisms, more detailed inflammatory cytokine profiles, and lifestyle factors) that could influence the results. Fifth, the threshold effect analysis for SII was based on a piecewise linear model and restricted to SII < 1,000 (*N* = 252); the inflection point estimate may be affected by sample size and model specification and requires validation in independent cohorts. This study also did not measure fluid or imaging biomarkers such as amyloid-β (Aβ), tau, or neurofilament light chain, and therefore could not directly examine the relationship between inflammatory indicators and AD pathological burden.

In summary, this study evaluated in parallel the associations of four composite inflammatory indicators—NLR, PLR, SII, and SIRI—with severe cognitive impairment in Chinese Han patients with AD. The results showed that NLR was independently and linearly associated with a higher risk of SCI, SII exhibited a threshold effect on SCI risk (with an inflection point at approximately 861.9), PLR showed no independent association after multivariable adjustment, and the association for SIRI weakened to borderline non-significance. These findings support an important role for systemic inflammation in AD progression and suggest that NLR and SII may serve as simple auxiliary biomarkers for assessing the severity of cognitive impairment in patients with AD; however, the shape and strength of the associations differ across composite inflammatory indicators, and further validation in larger, multicenter cohorts is warranted. Future multicenter, prospective studies with large sample sizes, combined with more refined neuroimaging and fluid biomarkers (such as Aβ, tau, and neurofilament light chain), are needed to further verify our findings and to explore the underlying pathophysiological mechanisms.

## Data Availability

The original contributions presented in the study are included in the article/Supplementary material, further inquiries can be directed to the corresponding author/s.
