# Supplementary material for: Association of systemic inflammation indices with severe cognitive impairment in Chinese Han patients with Alzheimer’s disease: a cross-sectional study of NLR, PLR, SII, and SIRI
Source: Front Aging Neurosci. 2026 Oct 27;18:1877189. doi: 10.3389/fnagi.2026.1877189 (PMC13748380; doi:10.3389/fnagi.2026.1877189)
Supplement: Supplementary file 1 [file Table_1.DOCX]

Table S1. Baseline characteristics of participants

| Variables | PLR | | | | P value |
| --- | --- | --- | --- | --- | --- |
|  | Q1 (37.04-103.49) | Q2 (103.85-140.57) | Q3(140.87-186.51) | Q4 (190.54-540.00) |  |
| N | 71 | 70 | 70 | 71 |  |
| Age (years old) | 76.15 ± 6.58 | 74.53 ± 7.41 | 76.16 ± 7.53 | 75.70 ± 8.93 | 0.55^a^ |
| Sex (n, %) |  |  |  |  | 0.54^b^ |
| Male | 34 (47.89%) | 25 (35.71%) | 29 (41.43%) | 30 (42.25%) |  |
| Female | 37 (52.11%) | 45 (64.29%) | 41 (58.57%) | 41 (57.75%) |  |
| Marital status (n, %) |  |  |  |  | 0.26^b^ |
| Unmarried | 0 (0.00%) | 3 (4.29%) | 0 (0.00%) | 1 (1.41%) |  |
| Married | 53 (74.65%) | 43 (61.43%) | 43 (61.43%) | 46 (64.79%) |  |
| Divorced | 0 (0.00%) | 1 (1.43%) | 0 (0.00%) | 1 (1.41%) |  |
| Widowed | 18 (25.35%) | 23 (32.86%) | 27 (38.57%) | 23 (32.39%) |  |
| Hypertension (n, %) |  |  |  |  | 0.33^b^ |
| No | 20 (28.17%) | 28 (40.00%) | 29 (41.43%) | 28 (39.44%) |  |
| Yes | 51 (71.83%) | 42 (60.00%) | 41 (58.57%) | 43 (60.56%) |  |
| Diabetes (n, %) |  |  |  |  | 0.01^b^ |
| No | 48 (67.61%) | 52 (74.29%) | 51 (72.86%) | 65 (91.55%) |  |
| Yes | 23 (32.39%) | 18 (25.71%) | 19 (27.14%) | 6 (8.45%) |  |
| NSAIDs (n, %) |  |  |  |  | 0.22^b^ |
| No | 65 (91.55%) | 58 (82.86%) | 61 (87.14%) | 66 (92.96%) |  |
| Yes | 6 (8.45%) | 12 (17.14%) | 9 (12.86%) | 5 (7.04%) |  |
| Antipsychotic use (n, %) |  |  |  |  | 0.82^b^ |
| No | 33 (46.48%) | 34 (48.57%) | 38 (54.29%) | 36 (50.70%) |  |
| Yes | 38 (53.52%) | 36 (51.43%) | 32 (45.71%) | 35 (49.30%) |  |
| Anti-AD drugs use (n, %) |  |  |  |  | 0.79^b^ |
| No | 29 (40.85%) | 32 (45.71%) | 32 (45.71%) | 35 (49.30%) |  |
| Yes | 42 (59.15%) | 38 (54.29%) | 38 (54.29%) | 36 (50.70%) |  |
| Smoking (n, %) |  |  |  |  | 0.98^b^ |
| No | 57 (80.28%) | 57 (81.43%) | 57 (81.43%) | 56 (78.87%) |  |
| Yes | 14 (19.72%) | 13 (18.57%) | 13 (18.57%) | 15 (21.13%) |  |
| Alcohol consumption (n, %) |  |  |  |  | 0.79^b^ |
| No | 51 (71.83%) | 53 (75.71%) | 54 (77.14%) | 56 (78.87%) |  |
| Yes | 20 (28.17%) | 17 (24.29%) | 16 (22.86%) | 15 (21.13%) |  |
| SCI (n, %) |  |  |  |  | 0.23^b^ |
| No | 37 (52.11%) | 38 (54.29%) | 40 (57.14%) | 29 (40.85%) |  |
| Yes | 34 (47.89%) | 32 (45.71%) | 30 (42.86%) | 42 (59.15%) |  |
| BMI (kg/m^2^) | 21.67 ± 3.44 | 22.69 ± 3.70 | 22.13 ± 3.33 | 20.70 ± 3.07 | 0.01^a^ |
| Years of education (years) | 0 (0-6) | 0 (0-6) | 6 (0-6) | 0 (0-6) | 0.08^c^ |
| Disease duration (years) | 3 (2-5) | 3 (2-5) | 2.5 (2-5) | 3 (2-5) | 0.51^c^ |
| MoCA score | 10.56 ± 4.89 | 10.40 ± 5.44 | 10.81 ± 5.55 | 9.65 ± 4.87 | 0.58^a^ |
| WBC (10^^^9/L) | 5.34 ± 1.56 | 5.40 ± 1.54 | 5.19 ± 1.48 | 5.35 ± 1.81 | 0.88^a^ |
| NEUT (10^^^9/L) | 3.02 ± 1.26 | 3.35 ± 1.30 | 3.45 ± 1.23 | 3.95 ± 1.70 | 0.001^a^ |
| LYMPH (10^^^9/L) | 1.83 ± 0.49 | 1.55 ± 0.50 | 1.27 ± 0.33 | 0.98 ± 0.26 | <0.001^a^ |
| MONO (10^^^9/L) | 0.36 ± 0.13 | 0.35 ± 0.12 | 0.35 ± 0.14 | 0.34 ± 0.10 | 0.95^a^ |
| EO (10^^^9/L) | 0.10 (0.07-0.15) | 0.11 (0.08-0.17) | 0.08 (0.05-0.15) | 0.09 (0.04-0.14) | 0.03^c^ |
| BASO (10^^^9/L) | 0.01 (0.01-0.02) | 0.01 (0.01-0.02) | 0.01 (0.01-0.02) | 0.01 (0.00-0.02) | 0.17^c^ |
| RBC (10^^^12/L) | 4.02 ± 0.53 | 3.97 ± 0.50 | 4.06 ± 0.52 | 3.98 ± 0.42 | 0.68^a^ |
| HGB (g/L) | 122.54 ± 19.12 | 121.99 ± 13.44 | 125.19 ± 15.48 | 121.86 ± 13.29 | 0.55^a^ |
| PLT (10^^^9/L) | 149.07 ± 41.58 | 190.70 ± 56.94 | 204.80 ± 50.19 | 243.41 ± 69.12 | <0.001^a^ |
| hs-CRP (mg/L) | 1.54 (0.94-3.68) | 1.82 (0.94-3.93) | 1.38 (0.74-2.69) | 1.91 (1.02-3.58) | 0.40^c^ |
| FBG (mmol/L) | 5.34 ± 1.97 | 5.67 ± 2.13 | 5.37 ± 1.23 | 5.16 ± 0.89 | 0.32^a^ |
| TG (mmol/L) | 1.05 (0.78-1.43) | 1.14 (0.73-1.52) | 0.99 (0.75-1.47) | 0.94 (0.76-1.23) | 0.50^c^ |
| TC (mmol/L) | 4.09 ± 0.95 | 4.34 ± 0.97 | 4.10 ± 0.90 | 4.20 ± 0.89 | 0.34^a^ |
| HDL-C (mmol/L) | 1.25 ± 0.36 | 1.19 ± 0.29 | 1.32 ± 0.74 | 1.26 ± 0.36 | 0.49^a^ |
| LDL-C (mmol/L) | 2.54 ± 0.78 | 2.83 ± 0.86 | 2.58 ± 0.75 | 2.67 ± 0.70 | 0.13^a^ |
| TSH (uIU/mL) | 2.02 (1.19-3.11) | 1.85 (1.18-3.08) | 1.70 (1.15-2.40) | 2.01 (1.29-2.95) | 0.41^c^ |
| FT3 (pmol/L) | 3.05 ± 0.44 | 3.03 ± 0.40 | 3.16 ± 0.60 | 2.93 ± 0.46 | 0.045^a^ |
| FT4 (pmol/L) | 1.17 ± 0.20 | 1.15 ± 0.21 | 1.22 ± 0.25 | 1.20 ± 0.26 | 0.23^a^ |
| T3 (nmol/L) | 1.02 ± 0.22 | 1.01 ± 0.22 | 1.10 ± 0.24 | 1.09 ± 0.40 | 0.13^a^ |
| T4 (nmol/L) | 7.90 ± 2.14 | 7.79 ± 2.08 | 8.65 ± 2.24 | 8.18 ± 2.20 | 0.09^a^ |

Note: The variables are presented as n (%) or the mean ± SD or median (quartile 1-quartile 3).

PLR: platelet/lymphocyte ratio; NSAIDs: nonsteroidal antiinflammatory drugs; AD: Alzheimer’s disease; SCI: severe cognitive impairment; BMI: body mass index; MoCA: Montreal Cognitive Assessment‌; WBC: white blood cell; NEUT: neutrophil; LYMPH: lymphocyte; MONO: monocytes; EO: eosinophil; BASO: basophil; RBC: red blood cell; HGB: hemoglobin; PLT: platelet; hs-CRP: hypersensitive C-reactive protein; FBG: fasting blood glucose;TG: triglycerides; TC: total cholesterol; HDL-C: high-density lipoprotein cholesterol; LDL-C: low-density lipoprotein cholesterol; TSH: thyroid stimulating hormone; FT3: free triiodothyronine; FT4: free thyroxine; T3: triiodothyronine; T4: thyroxine.

^a^ one-way ANOVA test, ^b^ χ2 test, and ^c^ Kruskal–Wallis H test
