# Supplementary material for: Association of systemic inflammation indices with severe cognitive impairment in Chinese Han patients with Alzheimer’s disease: a cross-sectional study of NLR, PLR, SII, and SIRI
Source: Front Aging Neurosci. 2026 Oct 27;18:1877189. doi: 10.3389/fnagi.2026.1877189 (PMC13748380; doi:10.3389/fnagi.2026.1877189)
Supplement: Supplementary file 2 [file Table_2.DOCX]

Table S2. Baseline characteristics of participants

| Variables | SII | | | | P value |
| --- | --- | --- | --- | --- | --- |
|  | Q1 (60.37-288.77) | Q2 (290.34-451.18) | Q3(457.12-668.85) | Q4 (676.32-2944.06) |  |
| N | 71 | 70 | 70 | 71 |  |
| Age (years old) | 76.32 ± 6.84 | 75.73 ± 7.35 | 74.77 ± 7.48 | 75.72 ± 8.86 | 0.69^a^ |
| Sex (n, %) |  |  |  |  | 0.89^b^ |
| Male | 30 (42.25%) | 27 (38.57%) | 29 (41.43%) | 32 (45.07%) |  |
| Female | 41 (57.75%) | 43 (61.43%) | 41 (58.57%) | 39 (54.93%) |  |
| Marital status (n, %) |  |  |  |  | 0.87^b^ |
| Unmarried | 1 (1.41%) | 1 (1.43%) | 2 (2.86%) | 0 |  |
| Married | 49 (69.01%) | 46 (65.71%) | 44 (62.86%) | 46 (64.79%) |  |
| Divorced | 0 | 1 (1.43%) | 0 | 1 (1.41%) |  |
| Widowed | 21 (29.58%) | 22 (31.43%) | 24 (34.29%) | 24 (33.80%) |  |
| Hypertension (n, %) |  |  |  |  | 0.38^b^ |
| No | 29 (40.85%) | 20 (28.57%) | 27 (38.57%) | 29 (40.85%) |  |
| Yes | 42 (59.15%) | 50 (71.43%) | 43 (61.43%) | 42 (59.15%) |  |
| Diabetes (n, %) |  |  |  |  | 0.29^b^ |
| No | 54 (76.06%) | 52 (74.29%) | 50 (71.43%) | 60 (84.51%) |  |
| Yes | 17 (23.94%) | 18 (25.71%) | 20 (28.57%) | 11 (15.49%) |  |
| NSAIDs (n, %) |  |  |  |  | 0.56^b^ |
| No | 64 (90.14%) | 62 (88.57%) | 59 (84.29%) | 65 (91.55%) |  |
| Yes | 7 (9.86%) | 8 (11.43%) | 11 (15.71%) | 6 (8.45%) |  |
| Antipsychotic use (n, %) |  |  |  |  | 0.63^b^ |
| No | 38 (53.52%) | 35 (50.00%) | 37 (52.86%) | 31 (43.66%) |  |
| Yes | 33 (46.48%) | 35 (50.00%) | 33 (47.14%) | 40 (56.34%) |  |
| Anti-AD drugs use (n, %) |  |  |  |  | 0.22^b^ |
| No | 32 (45.07%) | 28 (40.00%) | 39 (55.71%) | 29 (40.85%) |  |
| Yes | 39 (54.93%) | 42 (60.00%) | 31 (44.29%) | 42 (59.15%) |  |
| Smoking (n, %) |  |  |  |  | 0.85^b^ |
| No | 58 (81.69%) | 58 (82.86%) | 54 (77.14%) | 57 (80.28%) |  |
| Yes | 13 (18.31%) | 12 (17.14%) | 16 (22.86%) | 14 (19.72%) |  |
| Alcohol consumption (n, %) |  |  |  |  | 0.81^b^ |
| No | 52 (73.24%) | 53 (75.71%) | 56 (80.00%) | 53 (74.65%) |  |
| Yes | 19 (26.76%) | 17 (24.29%) | 14 (20.00%) | 18 (25.35%) |  |
| SCI (n, %) |  |  |  |  | 0.10^b^ |
| No | 36 (50.70%) | 38 (54.29%) | 42 (60.00%) | 28 (39.44%) |  |
| Yes | 35 (49.30%) | 32 (45.71%) | 28 (40.00%) | 43 (60.56%) |  |
| BMI (kg/m^2^) | 21.66 ± 3.53 | 22.02 ± 3.55 | 22.15 ± 3.31 | 21.35 ± 3.42 | 0.50^a^ |
| Years of education (years) | 0 (0-6) | 0 (0-6) | 0 (0-6) | 6 (0-6) | 0.65^c^ |
| Disease duration (years) | 3 (1.5-5) | 3 (2-5) | 3 (2-5) | 3 (2-5) | 0.90^c^ |
| MoCA score | 10.52 ± 4.71 | 10.43 ± 4.91 | 10.91 ± 5.81 | 9.56 ± 5.27 | 0.47^a^ |
| WBC (10^^^9/L) | 4.36 ± 1.11 | 4.93 ± 1.21 | 5.63 ± 1.49 | 6.37 ± 1.75 | <0.001^a^ |
| NEUT (10^^^9/L) | 2.27 ± 0.65 | 2.96 ± 0.83 | 3.72 ± 1.00 | 4.82 ± 1.54 | <0.001^a^ |
| LYMPH (10^^^9/L) | 1.66 ± 0.56 | 1.50 ± 0.50 | 1.39 ± 0.46 | 1.07 ± 0.33 | <0.001^a^ |
| MONO (10^^^9/L) | 0.30 ± 0.09 | 0.33 ± 0.08 | 0.38 ± 0.13 | 0.40 ± 0.15 | <0.001^a^ |
| EO (10^^^9/L) | 0.11 (0.07-0.15) | 0.10 (0.07-0.15) | 0.09 (0.06-0.16) | 0.09 (0.04-0.14) | 0.16^c^ |
| BASO (10^^^9/L) | 0.01 (0.01-0.02) | 0.01 (0.01-0.02) | 0.01 (0.01-0.02) | 0.01 (0.01-0.02) | 0.58^c^ |
| RBC (10^^^12/L) | 3.89 ± 0.52 | 3.98 ± 0.53 | 4.07 ± 0.46 | 4.10 ± 0.43 | 0.04^a^ |
| HGB (g/L) | 119.12 ± 18.81 | 121.54 ± 14.46 | 125.57 ± 13.27 | 125.34 ± 14.18 | 0.03^a^ |
| PLT (10^^^9/L) | 148.87 ± 48.82 | 188.36 ± 49.79 | 207.46 ± 45.67 | 243.30 ± 72.18 | <0.001^a^ |
| hs-CRP (mg/L) | 1.48 (0.83-2.70) | 1.87 (0.90-4.28) | 1.43 (0.74-2.62) | 1.98 (1.08-4.05) | 0.09^c^ |
| FBG (mmol/L) | 5.23 ± 2.13 | 5.47 ± 1.75 | 5.49 ± 1.43 | 5.35 ± 1.09 | 0.78^a^ |
| TG (mmol/L) | 0.98 (0.78-1.44) | 1.14 (0.70-1.56) | 1.08 (0.80-1.47) | 0.92 (0.70-1.27) | 0.39^c^ |
| TC (mmol/L) | 4.02 ± 0.87 | 4.21 ± 0.99 | 4.24 ± 0.98 | 4.25 ± 0.87 | 0.42^a^ |
| HDL-C (mmol/L) | 1.18 ± 0.28 | 1.31 ± 0.74 | 1.25 ± 0.36 | 1.28 ± 0.37 | 0.38^a^ |
| LDL-C (mmol/L) | 2.55 ± 0.79 | 2.68 ± 0.84 | 2.71 ± 0.79 | 2.67 ± 0.69 | 0.63^a^ |
| TSH (uIU/mL) | 1.82 (1.17-2.89) | 2.01 (1.29-2.91) | 1.77 (1.12-2.88) | 1.97 (1.29-2.92) | 0.93^c^ |
| FT3 (pmol/L) | 3.02 ± 0.43 | 3.08 ± 0.40 | 3.16 ± 0.62 | 2.91 ± 0.45 | 0.02^a^ |
| FT4 (pmol/L) | 1.15 ± 0.21 | 1.15 ± 0.19 | 1.22 ± 0.25 | 1.22 ± 0.25 | 0.09^a^ |
| T3 (nmol/L) | 0.99 ± 0.22 | 1.07 ± 0.20 | 1.07 ± 0.24 | 1.10 ± 0.41 | 0.09^a^ |
| T4 (nmol/L) | 7.58 ± 2.10 | 7.97 ± 1.90 | 8.55 ± 2.41 | 8.42 ± 2.17 | 0.03^a^ |

Note: The variables are presented as n (%) or the mean ± SD or median (quartile 1-quartile 3).

SII: systemic immune-inflammation index; NSAIDs: nonsteroidal antiinflammatory drugs; AD: Alzheimer’s disease; SCI: severe cognitive impairment; BMI: body mass index; MoCA: Montreal Cognitive Assessment‌; WBC: white blood cell; NEUT: neutrophil; LYMPH: lymphocyte; MONO: monocytes; EO: eosinophil; BASO: basophil; RBC: red blood cell; HGB: hemoglobin; PLT: platelet; hs-CRP: hypersensitive C-reactive protein; FBG: fasting blood glucose;TG: triglycerides; TC: total cholesterol; HDL-C: high-density lipoprotein cholesterol; LDL-C: low-density lipoprotein cholesterol; TSH: thyroid stimulating hormone; FT3: free triiodothyronine; FT4: free thyroxine; T3: triiodothyronine; T4: thyroxine.

^a^ one-way ANOVA test, ^b^ χ2 test, and ^c^ Kruskal–Wallis H test
