# Supplementary material for: Association of systemic inflammation indices with severe cognitive impairment in Chinese Han patients with Alzheimer’s disease: a cross-sectional study of NLR, PLR, SII, and SIRI
Source: Front Aging Neurosci. 2026 Oct 27;18:1877189. doi: 10.3389/fnagi.2026.1877189 (PMC13748380; doi:10.3389/fnagi.2026.1877189)
Supplement: Supplementary file 3 [file Table_3.DOCX]

Table S3. Baseline characteristics of participants

| Variables | SIRI | | | | P value |
| --- | --- | --- | --- | --- | --- |
|  | Q1 (0.13-0.50) | Q2 (0.50-0.76) | Q3(0.76-1.19) | Q4 (1.21-5.63) |  |
| N | 71 | 70 | 70 | 71 |  |
| Age (years old) | 75.65 ± 7.16 | 74.47 ± 7.37 | 76.30 ± 7.75 | 76.13 ± 8.29 | 0.49^a^ |
| Sex (n, %) |  |  |  |  | 0.02^b^ |
| Male | 23 (32.39%) | 23 (32.86%) | 37 (52.86%) | 35 (49.30%) |  |
| Female | 48 (67.61%) | 47 (67.14%) | 33 (47.14%) | 36 (50.70%) |  |
| Marital status (n, %) |  |  |  |  | 0.54^b^ |
| Unmarried | 1 (1.41%) | 1 (1.43%) | 2 (2.86%) | 0 |  |
| Married | 48 (67.61%) | 45 (64.29%) | 50 (71.43%) | 42 (59.15%) |  |
| Divorced | 0 | 1 (1.43%) | 1 (1.43%) | 0 |  |
| Widowed | 22 (30.99%) | 23 (32.86%) | 17 (24.29%) | 29 (40.85%) |  |
| Hypertension (n, %) |  |  |  |  | 0.54^b^ |
| No | 29 (40.85%) | 21 (30.00%) | 27 (38.57%) | 28 (39.44%) |  |
| Yes | 42 (59.15%) | 49 (70.00%) | 43 (61.43%) | 43 (60.56%) |  |
| Diabetes (n, %) |  |  |  |  | 0.48^b^ |
| No | 56 (78.87%) | 50 (71.43%) | 52 (74.29%) | 58 (81.69%) |  |
| Yes | 15 (21.13%) | 20 (28.57%) | 18 (25.71%) | 13 (18.31%) |  |
| NSAIDs (n, %) |  |  |  |  | 0.45^b^ |
| No | 66 (92.96%) | 60 (85.71%) | 60 (85.71%) | 64 (90.14%) |  |
| Yes | 5 (7.04%) | 10 (14.29%) | 10 (14.29%) | 7 (9.86%) |  |
| Antipsychotic use (n, %) |  |  |  |  | 0.13^b^ |
| No | 35 (49.30%) | 43 (61.43%) | 33 (47.14%) | 30 (42.25%) |  |
| Yes | 36 (50.70%) | 27 (38.57%) | 37 (52.86%) | 41 (57.75%) |  |
| Anti-AD drugs use (n, %) |  |  |  |  | 0.67^b^ |
| No | 32 (45.07%) | 29 (41.43%) | 36 (51.43%) | 31 (43.66%) |  |
| Yes | 39 (54.93%) | 41 (58.57%) | 34 (48.57%) | 40 (56.34%) |  |
| Smoking (n, %) |  |  |  |  | 0.20^b^ |
| No | 61 (85.92%) | 59 (84.29%) | 51 (72.86%) | 56 (78.87%) |  |
| Yes | 10 (14.08%) | 11 (15.71%) | 19 (27.14%) | 15 (21.13%) |  |
| Alcohol consumption (n, %) |  |  |  |  | 0.91^b^ |
| No | 54 (76.06%) | 55 (78.57%) | 53 (75.71%) | 52 (73.24%) |  |
| Yes | 17 (23.94%) | 15 (21.43%) | 17 (24.29%) | 19 (26.76%) |  |
| SCI (n, %) |  |  |  |  | 0.28^b^ |
| No | 39 (54.93%) | 32 (45.71%) | 41 (58.57%) | 32 (45.07%) |  |
| Yes | 32 (45.07%) | 38 (54.29%) | 29 (41.43%) | 39 (54.93%) |  |
| BMI (kg/m^2^) | 21.23 ± 3.44 | 22.12 ± 3.62 | 22.19 ± 3.06 | 21.63 ± 3.64 | 0.31^a^ |
| Years of education (years) | 0 (0-6) | 0 (0-6) | 5 (0-6) | 0 (0-6) | 0.80^c^ |
| Disease duration (years) | 3 (2-4.5) | 3 (2-5) | 3 (2-5) | 3 (2-5) | 0.61^c^ |
| MoCA score | 10.89 ± 5.03 | 9.51 ± 4.49 | 11.03 ± 5.47 | 9.99 ± 5.63 | 0.25^a^ |
| WBC (10^^^9/L) | 4.22 ± 1.05 | 4.98 ± 1.07 | 5.21 ± 1.23 | 6.86 ± 1.66 | <0.001^a^ |
| NEUT (10^^^9/L) | 2.18 ± 0.57 | 3.02 ± 0.65 | 3.46 ± 0.75 | 5.11 ± 1.47 | <0.001^a^ |
| LYMPH (10^^^9/L) | 1.64 ± 0.57 | 1.52 ± 0.49 | 1.31 ± 0.42 | 1.15 ± 0.43 | <0.001^a^ |
| MONO (10^^^9/L) | 0.26 ± 0.07 | 0.32 ± 0.07 | 0.36 ± 0.08 | 0.46 ± 0.15 | <0.001^a^ |
| EO (10^^^9/L) | 0.11 (0.06-0.15) | 0.10 (0.06-0.14) | 0.08 (0.06-0.15) | 0.10 (0.04-0.16) | 0.45^c^ |
| BASO (10^^^9/L) | 0.01 (0.01-0.02) | 0.02 (0.01-0.02) | 0.01 (0.01-0.02) | 0.01 (0.01-0.02) | 0.06^c^ |
| RBC (10^^^12/L) | 3.84 ± 0.53 | 3.96 ± 0.49 | 4.10 ± 0.45 | 4.14 ± 0.45 | 0.001^a^ |
| HGB (g/L) | 116.79 ± 19.28 | 122.40 ± 12.93 | 125.24 ± 12.95 | 127.14 ± 14.15 | <0.001^a^ |
| PLT (10^^^9/L) | 174.27 ± 58.85 | 197.77 ± 66.33 | 209.23 ± 73.77 | 206.87 ± 53.50 | 0.004^a^ |
| hs-CRP (mg/L) | 1.34 (0.61-2.25) | 1.60 (0.87-3.32) | 1.77 (1.08-3.22) | 2.32 (1.08-5.04) | 0.01^c^ |
| FBG (mmol/L) | 5.13 ± 1.89 | 5.42 ± 1.51 | 5.47 ± 1.39 | 5.52 ± 1.73 | 0.51^a^ |
| TG (mmol/L) | 1.02 (0.78-1.42) | 1.11 (0.76-1.50) | 1.17 (0.86-1.58) | 0.84 (0.70-1.18) | 0.004^c^ |
| TC (mmol/L) | 4.19 ± 0.93 | 4.11 ± 0.88 | 4.06 ± 0.94 | 4.35 ± 0.96 | 0.28^a^ |
| HDL-C (mmol/L) | 1.18 ± 0.28 | 1.34 ± 0.75 | 1.14 ± 0.24 | 1.36 ± 0.43 | 0.001^a^ |
| LDL-C (mmol/L) | 2.70 ± 0.81 | 2.58 ± 0.77 | 2.64 ± 0.80 | 2.69 ± 0.73 | 0.76^a^ |
| TSH (uIU/mL) | 1.82 (1.15-2.81) | 1.81 (1.20-2.68) | 1.92 (1.24-2.88) | 1.97 (1.11-3.33) | 0.82^c^ |
| FT3 (pmol/L) | 3.04 ± 0.38 | 2.95 ± 0.50 | 3.15 ± 0.59 | 3.03 ± 0.44 | 0.12^a^ |
| FT4 (pmol/L) | 1.15 ± 0.21 | 1.19 ± 0.25 | 1.20 ± 0.24 | 1.20 ± 0.22 | 0.53^a^ |
| T3 (nmol/L) | 0.98 ± 0.20 | 1.08 ± 0.41 | 1.08 ± 0.22 | 1.08 ± 0.23 | 0.10^a^ |
| T4 (nmol/L) | 7.75 ± 2.14 | 7.86 ± 2.36 | 8.25 ± 2.19 | 8.65 ± 1.92 | 0.06^a^ |

Note: The variables are presented as n (%) or the mean ± SD or median (quartile 1-quartile 3).

SIRI: systemic inflammation response index; NSAIDs: nonsteroidal antiinflammatory drugs; AD: Alzheimer’s disease; SCI: severe cognitive impairment; BMI: body mass index; MoCA: Montreal Cognitive Assessment‌; WBC: white blood cell; NEUT: neutrophil; LYMPH: lymphocyte; MONO: monocytes; EO: eosinophil; BASO: basophil; RBC: red blood cell; HGB: hemoglobin; PLT: platelet; hs-CRP: hypersensitive C-reactive protein; FBG: fasting blood glucose;TG: triglycerides; TC: total cholesterol; HDL-C: high-density lipoprotein cholesterol; LDL-C: low-density lipoprotein cholesterol; TSH: thyroid stimulating hormone; FT3: free triiodothyronine; FT4: free thyroxine; T3: triiodothyronine; T4: thyroxine.

^a^ one-way ANOVA test, ^b^ χ2 test, and ^c^ Kruskal–Wallis H test
